# The GoodNight study—online CBT for insomnia for the indicated prevention of depression: study protocol for a randomised controlled trial

**DOI:** 10.1186/1745-6215-15-56

**Published:** 2014-02-13

**Authors:** John A Gosling, Nick Glozier, Kathleen Griffiths, Lee Ritterband, Frances Thorndike, Andrew Mackinnon, Kanupriya Kalia Hehir, Anthony Bennett, Kylie Bennett, Helen Christensen

**Affiliations:** 1 Centre for Mental Health Research, The Australian National University, Building 63, Eggleston Road, Canberra, ACT 0200, Australia; 2 Psychiatry, Central Clinical School and Brain and Mind Research Institute, University of Sydney Sydney, Australia; 3 Department of Psychiatry and Neurobehavioural Sciences, University of Virginia Health System, Charlottesville, USA; 4 Orygen Youth Health Research Centre, University of Melbourne, Melbourne, Australia; 5 Black Dog Institute, University of New South Wales, Sydney, Australia

## Abstract

**Background:**

Cognitive Behaviour Therapy for Insomnia (CBT-I) delivered through the Internet is effective as a treatment in reducing insomnia in individuals seeking help for insomnia. CBT-I also lowers levels of depression in this group. However, it is not known if targeting insomnia using CBT-I will lower depressive symptoms, and thus reduce the risk of major depressive episode onset, in those specifically at risk for depression. Therefore, this study aims to examine whether Internet delivery of fully automated self-help CBT-I designed to reduce insomnia will prevent depression.

**Method/design:**

A sample of 1,600 community-dwelling adults (aged 18–64), who screen positive for both subclinical levels of depressive symptoms and insomnia, will be recruited via various media and randomised to either a 9-week online insomnia treatment programme, Sleep Healthy Using The internet (SHUTi), or an online attention-matched control group (HealthWatch). The primary outcome variable will be depression symptom levels at the 6-month post-intervention on the Patient Heath Questionnaire-9 (PHQ-9). A secondary outcome will be onset of major depressive episodes assessed at the 6-month post-intervention using ‘current’ and ‘time from intervention’ criteria from the Mini International Neuropsychiatric Interview.

**Discussion:**

This trial is the first randomised controlled trial of an Internet-based insomnia intervention as an indicated preventative programme for depression. If effective, online provision of a depression prevention programme will facilitate dissemination.

**Trial registration:**

Australian New Zealand Clinical Trials Registry (ANZCTR), Registration number: ACTRN12611000121965.

## Background

Depression is a leading cause of disability throughout the world. In the US, depression is estimated to represent a total lifetime economic cost for all those affected of 2.1 trillion dollars and at an individual level a lifetime loss of $300,000 per family affected [1]. Globally, depression is the leading cause of disease burden as measured by years lost to disability (YLD), representing roughly 20 million years and 31 million years lost because of disability annually for males and females respectively [2].

There is now considerable evidence that major depressive disorder (MDD) can be prevented [3]. However, the best methods to prevent depression are still debated [4]. Although cognitive behaviour therapy as an intervention is strongly supported [5], it is not clear whether interventions to prevent depression should take an indicated, selective or universal approach [3]. The present trial (The GoodNight Study) aims to prevent depression by reducing insomnia in individuals with depression symptoms, although not meeting criteria for MDD, and thus represents an indicated prevention trial of depression using an intervention designed to improve sleep.

In this study protocol paper, the development of the GoodNight Study is described. We provide a description of the relationship between insomnia and MDD to provide a rationale for our approach, then outline the methods, research design and analysis strategy, followed by a brief discussion of the potential implications of the study.

### The relationship between insomnia and depression

The relationship between insomnia and depression has received considerable attention in the past decade. Focus has shifted from the view that insomnia is a symptom, or sequala, of depression to one in which it is conceptualised as a related but not totally dependent phenomenon [6]. Supporting this, insomnia is commonly unresponsive to otherwise successful treatment for depression, being the most common residual symptom following completion of either pharmacological or psychological depression treatment [7,8], and the timing of insomnia onset more often than not precedes the onset of a depressive episode [9]. Insomnia is a strong independent risk factor for both initial episodes of MDD and episodic recurrence [10]. All major longitudinal North American psychiatric epidemiological studies confirm this finding: the Epidemiological Catchment Area study [11], the National Comorbidity Study [12], Johns Hopkins Precursor study [13], the Stirling County Study 9 [14] and those from Europe [15] and elsewhere [16]. Work from the Zurich Cohort [17] and other multiply sampled studies [18] has shown that this effect is independent of previous episodes of MDD and subclinical symptoms of depression. Recent evidence even suggests that insomnia treatment can positively affect the treatment course and outcome for non-sleep aspects for a range of psychiatric disorders [19]. Self-help therapy for insomnia is also associated with improvements in depression and anxiety [20-24]. While not demonstrating a causal association, this set of findings suggests that the treatment of insomnia may lower depressive symptoms and prevent the development of a first or recurrent episode of major depressive disorder [25].

Cognitive Behavioural Therapy for Insomnia (CBT-I) is amongst the most effective treatments for insomnia [26]; it is as effective as pharmacotherapy in the long term, with the added benefit of improvements persisting beyond cessation of treatment [27] and without the possible side effects. Recent evidence also suggests that CBT-I can have the benefit of reducing comorbid depression, decreasing both depression-related sleep-disturbance [28,29] and depressive symptomatology [19,29]. There is preliminary evidence suggesting that Internet-based CBT-I in individuals reporting with insomnia also results in reduced levels of depressive symptoms, in addition to significantly reducing insomnia [30]. However, the question of whether major depressive episodes can be *prevented* by treating insomnia has not yet been answered.

The current trial uses a cognitive behaviour therapy (CBT) intervention for sleep, delivered as a web-based programme, SHUTi [31]. The choice of an online application such as SHUTi was motivated by the observation that Internet programmes can offer the intervention to a large number of individuals [32]. Online interventions, if effective, have wide-scale reach and can be disseminated for little cost [33], making them ideal for prevention at a population level. They are also often preferred over traditional face-to-face methods for their ability to provide anonymity, convenience, and ability to be used at home [34].

Sixteen hundred community-dwelling adults from Australia will be randomised to one of two conditions: SHUTi, an online Cognitive Behavioural Therapy intervention for Insomnia, shown to be effective in decreasing insomnia severity and improving sleep efficiency in two US trials [31,35], or HealthWatch, an attention-matched control condition used in previous RCTs run through the Centre for Mental Health Research, ANU [36,37].

## Methods/design

### Sample and recruitment

A total of 1,600 community-dwelling adults from across Australia with insomnia and subclinical levels of depression will be recruited using advertisements placed on popular online social networking sites [38,39], through sleep and/or mental health associations and foundations, through Google Advertising [38], and through media releases and newspaper articles. We expect that those at risk of insomnia or experiencing insomnia will select to click on the advertisement, where they will find information about the trial. Participants will then proceed to a screening page to determine eligibility for the study.

### Screening

At screening, respondents indicate whether they are aged between 18 and 64, have a valid email address and telephone number, and live in Australia. If those interested are eligible (see below for criteria), they will complete the Bergen Insomnia Scale (BIS) [40] and the Patient Health Questionnaire 9 (PHQ-9) [41]. If insomnia and subclinical depression are indicated (see below), respondents will be provided with a study information sheet and invited to give informed consent for a telephone call from trial staff to check eligibility and subsequently to take part in the study if ultimately found eligible. This telephone-based diagnostic interview will then be conducted by an experienced telephone interviewer. Sections of the Mini International Neuropsychiatric Interview (MINI) [42] relating to ‘current’ and ‘lifetime’ depression, current mania, panic disorder, social phobia, and generalised anxiety as well as a modified version of the Morin Insomnia Interview used in the original trial of SHUTi [43] will be administered. Potential participants must then complete ten daily sleep diary entries within a 2-week period prior to randomisation.

### Inclusion/exclusion criteria

Initial eligibility criteria are age 18 to 64; Bergen Insomnia Scale score of 3 or above on at least one of the first four items and a score of 3 or above on at least one of the last two items; and a score greater than 4 but less than 20 on the PHQ-9. Participants will also be excluded if their bedtime is outside of the hours of 8:00 p.m. to 2:00 a.m. more than twice a week, notwithstanding if this is due to insomnia. Similarly, participants will be excluded if their rising time is outside of the hours of 4:00 a.m. to 10:00 a.m. more than twice a week, again notwithstanding if this is due to insomnia. These stipulations will eliminate respondents who are shift workers, carers of young children, or who have other commitments that are likely to interfere with their ability to adhere to a regular nighttime sleep pattern. Participants will also be excluded if they are pregnant; do not have reliable Internet access at home or at work; cannot comfortably read English; have ever been diagnosed with psychosis, schizophrenia, or bipolar mood disorder by a psychiatrist; or are currently undergoing a non-drug treatment programme for insomnia with a health professional. Potential participants will also need to consent to a telephone-based diagnostic interview and supply at least one telephone number and one active email address.

In the diagnostic telephone interview, participants must meet criteria for insomnia on Morin’s modified diagnostic insomnia interview. These criteria include: (a) sleep-onset insomnia and/or sleep maintenance insomnia (>30 min for at least 3 nights/week), (b) insomnia symptoms lasting at least 1 month, and (c) sleep disturbance (or associated daytime fatigue) causing significant distress or impairment in social, occupational, or other areas of functioning. Participants will be excluded on the basis of this interview if they have any other sleep disorder, have had any medication changes in the preceding 3 months, have a medical illness accounting for their sleep disturbance, or are currently receiving formal psychological treatment for insomnia. On the MINI [42], participants must fail to meet 2-week diagnostic criteria for MDD and lifetime criteria for bipolar mood disorder. Participants with suicidal plans or attempts in the previous 2 weeks will also be excluded. These participants will be contacted by a clinical psychologist and provided with appropriate referral information.

### Experimental design and conditions

#### Experimental condition—online cognitive behavioural therapy for insomnia: SHUTi

The intervention condition utilises SHUTi, an online insomnia intervention [43] based on Cognitive Behaviour Therapy for Insomnia (CBT-I). SHUTi incorporates six modules or Cores: introduction/overview, sleep restriction and stimulus control 1sleep restriction and stimulus control 2, cognitive restructuring, sleep hygiene, and relapse prevention.

**Figure 1:**
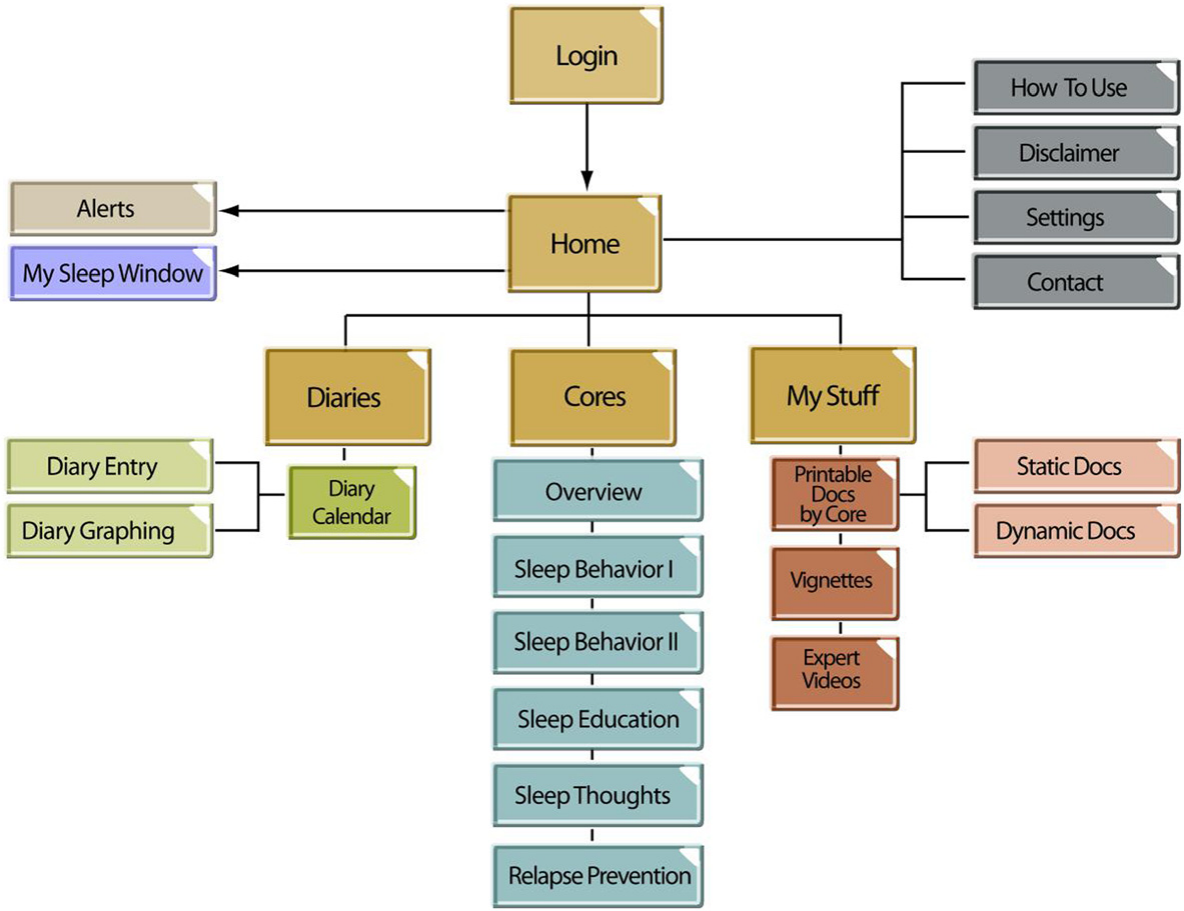
**SHUTi framework.** Shows a schematic of the Sleep Healthy Using the Internet (SHUTi) framework.

Once individuals have been deemed eligible and provided access to SHUTi, they are presented with a personalised homepage when first logged in. This homepage provides information to the user regarding their progress, tasks to complete (including alerts about sleep diary completion and Core assignments), a tailored Sleep Window, and options for navigating the programme. There is an Information Centre on the left of the screen, allowing access to the main sections of the site: Home, Diaries, Cores, and My Stuff (where users can access both static and dynamically generated printable documents from each Core as well as link to stories and expert videos in the intervention). Alerts as to the progress and upcoming assignments are also displayed. Sleep diaries and a diary calendar can also be accessed through the Information Centre tabs. Tabs entitled “How to use”, “Contact Us”, and “Disclaimer” are accessible at any time. “How to use” provides a brief tutorial to the user on how best to use the programme. “Contact Us” and “Disclaimer” provide contact details and a reminder that the contents of SHUTi are educational only and are not intended to replace the advice of a physician. See Figure 1 for a detailed schematic of the SHUTi framework.

Participants are encouraged to complete sleep diaries [44] on a daily basis throughout their use of SHUTi. SHUTi relies on sleep diary data that are entered by the user to track progress and to tailor programme recommendations. The sleep diary consists of 11 questions about the user’s sleep, including the hour of bedtime, minutes to fall asleep, number of night time awakenings, duration of night time awakenings, hour of awakening in the morning, hour of rising (getting out of bed) in the morning, length of daytime naps, amount of alcohol consumed, and a description of any sleep aids that were used. Users are also asked to rate the quality of their sleep on a 5-point scale from very poor to very good. Information recorded through daily sleep diaries is then used by the SHUTi system to calculate a user’s sleep efficiency and to assign, on a weekly basis, personalised sleep windows, i.e. the period of time in which the participant is instructed to sleep. Users are told that they should not sleep outside of this sleep window. In the 7 days between each Core, users must enter five of seven diaries to be assigned a new sleep window. This essential component of the programme restricts the hours during which a user is instructed to sleep/stay in bed in an effort to increase sleep efficiency. Algorithms have been developed to determine whether the sleep window should be increased, decreased, or maintained. Users select their rising time and the programme assigns a final sleep window (minimum of 5 h). Users are also able to view a flexible graphical representation of their sleep data from within the Diary section of the system.

The SHUTi system operates on a graduated framework, with users gaining access to a new Core 1 week after the previous Core has been completed. In addition to feedback about sleep pattern and tailored sleep windows, each Core acts as an online analogue for the weekly sessions typically used when delivering CBT in a face-to-face format. Each Core follows the same general structure: review of the previous weeks’ homework with feedback, review of diary data and revision to the Sleep Window, a summary of what will be learned in the current Core and why this information is important, new primary content, a homework screen that lists suggestions for how to improve sleep over the coming week, and a summary screen that provides a review of the main points presented in the Core. Both the homework and summary screens can be printed directly from the Core, as well as from the My Stuff section of the programme, which contains all static and dynamic documents, as well as all stories and videos, from each Core maintained in a convenient location. The main content screens for each Core address a unique aspect of insomnia treatment through a variety of interactive features, including a variety of ‘pop up’ buttons (e.g., myth/reality buttons or “learn more” buttons that provide in-depth information about a topic), animations that provide a topic visual to enhance comprehension, quizzes to test a user’s knowledge and impart additional information, and a series of vignettes or stories presented in both high-quality video and pictorially.

In addition to the tailoring in each Core, the programme also employs personalised goal-setting exercises. For example, at the beginning of the programme, users are asked to set specific targets for different aspects of their sleep including sleep onset latency, number of nighttime awakenings, and time spent awake in the middle of the night. At the end of the study, they are asked to re-evaluate their goals and to track their progress towards meeting them. Further tailoring occurs throughout the SHUTi programme in the form of various symptom checklists and homework assignments. Additional details of SHUTi can be found in Thorndike et al. [43].

#### Attention-matched control

The attention-matched control condition uses a 9-week version of HealthWatch, an Internet-delivered interactive lifestyle website with no specific mental health or sleep-related content. In previous trials HealthWatch [36,37,45] was not associated with reductions in depression symptoms [37]. Modules within HealthWatch are comprised of information pertaining to environmental health, nutrition myths, heart health, activity, medication, the effects of temperature, oral health, blood pressure and cholesterol, calcium, and back pain, as well as surveys about each of these topics each week.

### Assessments

Assessments are completed at pretest; immediate post-test; and 6-, 12-, and 18-month follow-up. Pretest assessment includes demographic information; outcome measures pertaining to insomnia (Insomnia Severity Index-ISI [46]), depression symptomatology (PHQ-9 [41]), anxiety (Generalised Anxiety Disorder, 7 items-GAD-7 [47]), suicidal ideation (from the Psychiatric Symptom Frequency scale-PSF [48]), disability (World Health Organisation Disability Assessment Schedule, 12 items-WHODAS-12 [49]), physical health and physical activity (International Physical Activity Questionnaire, Short Form-IPAQ SF [50]), attitudes toward help-seeking (Actual Help Seeking Questionnaire-AHSQ [51]/General Help Seeking Questionnaire-GHSQ [52]), and potential correlates of treatment outcome such as dysfunctional beliefs about sleep (Dysfunctional Beliefs About Sleep measure-DBAS [53]), sleep-threat monitoring (Sleep-Associated Monitoring Index-SAMI [54]), condition preference, and perceived need for treatment. Participants’ cognitive functioning will also be measured during the telephone-based diagnostic interview (Brief Test of Adult Cognition via Telephone-BTACT, Speed of Processing Subscale [55]). During the intervention phase of the study, participants will complete ongoing assessments on the ISI [46,56] and, to measure ongoing depression and anxiety symptomatology, the PHQ-9 [41] and GAD-7 [47] respectively at weeks 2, 4, 6, and 8.

At post-test, participants will complete an online questionnaire similar to that at pretest (see Table 1) in addition to receiving a telephone call in which they will again be administered the MINI [42] and the BTACT [57].

**Table 1:** Assessment measures in the GoodNight study

	**Online screener**	**Screening telephone interview**	**Pre-test survey**	**During intervention**	**Immediate post-test**	**6-, 18-month f/u**	**12-month f/u**
**PHQ**-**9**^**1**^	X		X	X	X	X	X
**BIS**^**2**^	X						
**Secondary eligibility criteria**	X						
**MINI**^**3**^		X			X	X	
**Adapted Morin insomnia interview**		X					
**BTACT**^**4**^		X			X	X	
**Demographics**			X				
**Condition preference**			X				
**Perceived need for treatment**			X				
**GAD-7**^**5**^			X	X	X	X	X
**PSF**^**6 **^**(suicidal ideation items)**			X		X	X	X
**DBAS**-**16**^**7**^			X		X	X	X
**SAMI**^**8 **^**(shortened)**			X		X	X	X
**IPAQ SF**^**9**^			X		X	X	X
**AHSQ/GHSQ**^**10**^			X		X	X	X
**CSRI**^**11**^			X		X	X	X
**SF12**^**12**^			X		X	X	X
**WHODAS 12**^**13**^			X		X	X	X
**ISI**^**14**^			X	X	X	X	X
**Sleep diaries**			X	X (SHUTi)	X	X	X
**Adherence questionnaire**					X		
**Internet evaluation and utility**					X		

^1^Patient Health Questionnaire-9 item.

^2^Bergen Insomnia Scale.

^3^Mini-International Neuropsychiatric Interview.

^4^Brief Test of Adult Cognition by Telephone.

^5^General Anxiety Disorder-7 item.

^6^Psychiatric Symptom Frequency scale.

^7^Dysfunctional Beliefs About Sleep-16 item.

^8^Sleep-Associated Monitoring Index.

^9^International Physical Activity Questionnaire-Short Form.

^10^Actual Help Seeking Questionnaire/General Help Seeking Questionnaire.

^11^Client Service Receipt Inventory.

^12^Short Form-12 item.

^13^World Health Organisation Disability Assessment Schedule-12 item.

^14^Insomnia Severity Index.

At 6-, 12-, and 18-month follow-up, participants will again complete online assessments and at 6- and 18-month follow-up will receive a telephone call in which they will be administered the MINI and the BTACT.

### Study hypotheses

#### Primary outcome

Participants receiving the SHUTi intervention will have lower levels of depression symptomatology on the PHQ-9, controlling for pretest depression levels, at 6 months compared to participants in the attention-matched control condition.

#### Secondary outcomes

The incidence of depressive episodes in the period since pre-test at 6-month follow-up will be lower in the SHUTi intervention arm compared to the control condition. This will be measured using the MINI. Related outcomes include a lower incidence of depressive episodes at 18-month follow-up. SHUTi participants will also have lower PHQ-9 scores at post-test, 12- and 18- month follow-up relative to controls, along with lower levels of insomnia on the Insomnia Severity Index, lower anxiety levels as measured by the GAD-7 [47] scores, lower suicidal ideation as measured using the PSF suicide items [48], and lower sleep and depression-related disability (i.e., impact on day-to-day functioning) as measured by the WHODAS-12 [58]. Compared to the control group, SHUTi intervention participants will also demonstrate increases in cognitive functioning, as measured by the BTACT [58], and increases in help seeking, as measured using the GHSQ [51] and AHSQ [52].

### Cost effectiveness

The incremental effectiveness will be determined by the difference in change scores in the intervention and control groups on the depression measure PHQ-9 [41]. The generic effectiveness will be measured using HRQOL from the SF-12 (Short Form 12) [59] and the WHODAS-12 (World Health Organization Disability Assessment Schedule, 12-item version) [58].

Costs will be measured from a health service and productivity perspective. Health service usage will be ascertained through:

i). self-report measurement (using the Client Service Receipt Inventory, CSRI) [60] and service use calculated on the basis of standard service costs, and

ii). data from the Australian Medicare Benefits Scheme/Pharmaceutical Benefits Scheme will be matched up with known costs of such services,

iii). extra out-of-pocket expenses associated with accessing these services, and

iv). costs associated with production loss relating to days out of role due to disability.

### Power

While the primary outcome variable of the study is a participant’s score on the PHQ-9 (a continuous measure), power calculations were based on rates of caseness for MDD as a larger sample is required to be able to detect changes in this secondary, but important, binary outcome. Estimates of power to detect differences in the proportion of probable cases of MDD as indicated by the PHQ-9 at 6-month follow-up were based on the estimated rate of onset of MDD in our target group and the anticipated effect of the SHUTi intervention. Based upon data from a subsample of participants in the PATH Through Life study [61] with comparable initial depression scores, and adjusting for the length of observation (6 months for this study vs. 4 years for PATH), approximately 15% of the no-intervention group with subclinical levels of depressive symptoms is expected to progress to probable MDD (a score of 15 or above on the PHQ-9, classified as moderately severe to severe depression and indicative of probably MDD [62]) within 6 months. We expect, based on data from previous research utilising SHUTi, a reduction of 40% of incident episodes in the treated group. For a total sample of 80% power, with a 40% reduction in risk in a sample with a pre-test incidence rate of 15%, a total sample size of 972 is needed. Economies of scale inherent in our automatised trial implementation should allow us to recruit a total sample of 1,600. This will maintain power if the incidence rate proves to be only 10% (total sample needed = 1,526) or if recruitment rates are less than anticipated. The target sample size will also maintain a power of 80% if the efficacy of the intervention is as low as 30%.

### Random allocation procedure

Randomisation will occur immediately following completion of the pre-assessment sleep diary phase using automated procedures integrated into the trial management software. Participants will be stratified by age and gender.

### Statistical analysis

Primary analyses will be undertaken on an intent-to-treat (ITT) basis, including data for all participants who were randomised, irrespective of their level of adherence to the intervention or whether they withdrew at any point in time. Participants with missing data will be accommodated using linear and generalised mixed-model repeated measure (MMRM) models. Transformations, including dichotomisation or other categorisation, will be undertaken as necessary to meet distributional assumptions and to accommodate outlying observations.

### Ethics approval

The GoodNight Study has received ethics approval from the Australian National University Human Research Ethics Committee, protocol no. 2011/041.

## Discussion

The prevention of depression is crucial if burden of disease attributable to this condition is to be reduced substantially. There are a number of novel features to the present trial. It will be the first trial targeting insomnia as a means of preventing the development of MDD. The trial is also novel in that it attempts to attract those at risk of depression to seek intervention by focussing on a condition that may not be stigmatising. Finally, it uses an online intervention that is capable of being disseminated at low cost to the community should it be found that SHUTi is effective. With follow-up time points at 6, 12, and 18 months post-intervention, the long-term impact of this intervention on depression, as well as its impact on secondary outcomes such as anxiety, suicidal ideation, disability, cognition, and help-seeking, can be assessed. A thorough analysis of potential correlates of intervention outcome will also allow for subsequent refinement and tailoring of the programme. Use of cost-effectiveness analyses will further provide information about the feasibility and economic benefit of implementing such an indicated prevention programme on a population level.

## Trial status

Recruitment to the GoodNight Study commenced in April 2013. The final participants are expected to complete their 18-month follow-up assessments in late 2015.

## Abbreviations

CBT-I: Cognitive-behavioural therapy for insomnia; SHUTi: Sleep health using the internet; RCT: Randomised controlled trial; MDD: Major depressive disorder; YLD: Years lost to disability; ANU: Australian National University; ANZCTR: Australian New Zealand Clinical Trials Registry; BIS: Bergen insomnia scale; PHQ-9: Patient health questionnaire-9 item; MINI: Mini international neuropsychiatric interview; GAD-7: Generalised anxiety scale-7 item; PSF: Psychiatric symptom frequency; WHODAS-12: World Health Organisation Disability Assessment Schedule, 12 item; IPAQ SF: International physical activity questionnaire, short form; AHSQ: Actual help-seeking questionnaire; GHSQ: General help-seeking questionnaire; DBAS: Dysfunctional beliefs about sleep measure; SAMI: Sleep-associated monitoring index; BTACT: Brief test of adult cognition via telephone; ISI: Insomnia severity index; CSRI: Client service receipt inventory; PATH: Path through life study; ITT: Intention to treat; MMRM: Mixed-model repeated measures.

## Competing interests

Drs. Ritterband and Thorndike have equity ownership in BeHealth Solutions, LLC, a company developing and making available products related to the research reported in this publication. Specifically, BeHealth Solutions, LLC, has licensed the SHUTi programme and the software platform on which it was built from the University of Virginia. The terms of this arrangement have been reviewed and approved by the University of Virginia in accordance with its conflict of interest policy.

## Authors’ contributions

HC conceived of the idea for this study, obtained funding, and is Chief Investigator A on the study; JG is Trial Manager for this study, contributed to the overall design, and drafted the manuscript; NG and KG are Chief Investigators on the project and contributed to the design of the study as well as contributing to editing and refinement of the manuscript; LR and FT are Associate Investigators on the study, developed the active intervention programme, have contributed to the design of the study, and contributed to editing and refinement of the manuscript; AM is a Chief Investigator on this study, is a biostatistician for the study and therefore ran power calculations and contributed regarding the measures to be included, the statistical analyses to be performed, and the overall research design; KKH was previously co-Trial Manager on the study, contributed to the study design, and helped edit the manuscript; AB and KB are Associate Investigators on the study, are responsible for of the IT implementation of the trial and therefore gave input regarding the design and flow of the trial, as well as contributing to the editing and refinement of the manuscript. All authors read and approved the final manuscript.
